# Neuraminidase Antibodies and H5N1: Geographic-Dependent Influenza Epidemiology Could Determine Cross-Protection against Emerging Strains

**DOI:** 10.1371/journal.pmed.0040212

**Published:** 2007-06-26

**Authors:** Jesus F Bermejo-Martin, David J Kelvin, Yi Guan, Honglin Chen, Pilar Perez-Breña, Inmaculada Casas, Eduardo Arranz, Raul O de Lejarazu

We have read with great interest the work of Sandbulte et al. recently published in your journal [1]. In this article, the authors provide evidence for the existence of cross-immunity between the neuraminidase of H5N1 viruses and that of endemic human H1N1 viruses. Age may be an important determining factor in the development of cross-immunity: younger people, having a shorter history of H1N1 exposure, may be disproportionately susceptible to H5N1 infection.

We would like to highlight the influence of the geographic-dependent epidemiological behaviour of influenza in the development of cross-immunity. While Europe, the United States, and northern Asia experience regular outbreaks of influenza each year, (“seasonal influenza”), influenza in tropical regions such as southern China, Vietnam, and Indonesia tends to be year-round (“non-seasonal” influenza). In consequence, the probability of exposure to influenza A in these regions persists throughout the entire year. Repetitive contacts with influenza wild viruses could promote the development of cross-immunity against different viral strains. Even more, it could represent a fortuitous mechanism for developed natural protection by the close and persistent exposure of the immune system to influenza wild viruses in regions known for being an important source of emergent viruses, like southern China.

Results from Sandbulte et al. show that antibodies play a dominant role in cross-protection. The authors underscore the possible benefit of seasonal influenza vaccination for human populations faced with the threat of pandemic H5N1 influenza. This idea deserves careful analysis. The main group at risk for severe complications of seasonal flu are people older than 65. In Western countries, this population is recommended to receive annual vaccinations. Generally speaking, elder vaccination rates in tropical countries are far lower than those in Western countries. Even with the low annual vaccination rate in elders, H5N1 infection is observed mostly in young people. The existence of sub-clinical or asymptomatic infections in elderly people cannot be ruled out, but the reason why there are no described clinical cases of H5N1 in people older than 40 years is currently unknown. An age-dependent differential distribution of avian-type receptors in the upper respiratory tract could be a possible explanation. On the other hand, Tumpey et al. [2] demonstrated that mucosal (but not parenteral) challenges with inactivated or live H3N2 virus protect against H5N1 infection in mice. These results could have a relevant consequence: does contact with circulating influenza A via the respiratory tract confer a higher degree of cross-protection than parenteral exposure to vaccines?

In conclusion, the non-seasonal epidemiological behaviour of influenza in tropical countries could dramatically influence the development of naturally induced cross-immunity against different influenza strains and diminish the risk of severe disease from new emergent strains in elderly people living in these countries. The apparent lack of H5N1 cases in the elderly may be the result of continued exposure to circulating non-seasonal influenza A via mucosal epithelium in the respiratory tract. Vaccination via the mucosal route could be a more efficient way to provide cross-protection against future pandemic strains than vaccination via the parenteral route. In this hypothetical scenario, Western countries would be under-protected.
